# Mirror Visual Feedback Combining Vibrotactile Stimulation Promotes Embodiment Perception: An Electroencephalogram (EEG) Pilot Study

**DOI:** 10.3389/fbioe.2020.553270

**Published:** 2020-10-26

**Authors:** Li Ding, Jiayuan He, Lin Yao, Jinyang Zhuang, Shugeng Chen, Hewei Wang, Ning Jiang, Jie Jia

**Affiliations:** ^1^Department of Rehabilitation Medicine, Huashan Hospital, Fudan University, Shanghai, China; ^2^Department of Systems Design Engineering, Faculty of Engineering, University of Waterloo, Waterloo, ON, Canada; ^3^Department of Neurobiology, NHC and CAMS Key Laboratory of Medical Neurobiology, School of Brain Science and Brain Medicine, and the MOE Frontier Science Center for Brain Research and Brain-Machine Integration, Zhejiang University School of Medicine, Hangzhou, China

**Keywords:** mirror visual feedback, embodiment perception, electroencephalogram (EEG), vibrotactile stimulation, neurorehabilitation

## Abstract

As one determinant of the efficacy of mirror visual feedback (MVF) in neurorehabilitation, the embodiment perception needs to be sustainable and enhanced. This study explored integrating vibrotactile stimulation into MVF to promote the embodiment perception and provide evidence of the potential mechanism of MVF. In the experiment, the participants were instructed to keep their dominant hand still (static side), while open and close their non-dominant hand (active side) and concentrate on the image of the hand movement in the mirror. They were asked to tap the pedal with the foot of the active side once the embodiment perception is generated. A vibrotactile stimulator was attached on the hand of the active side, and three conditions were investigated: no vibration (NV), continuous vibration (CV), and intermittent vibration (IV). The effects were analyzed on both objective data, including latency time (LT) and electroencephalogram (EEG) signals, and subjective data, including embodiment questionnaire (EQ). Results of LT and EQ suggested a stronger subjective sense of embodiment under the condition of CV and IV, comparing with NV. No significant difference was found between CV and IV. EEG analysis showed that in the hemisphere of the static side, the desynchronization of CV and IV around the central-frontal region (C3 and F3) in the alpha band (8–13 Hz) was significantly prominent compared to NV, and in the hemisphere of the active side, the desynchronization of three conditions was similar. The network analysis of EEG data indicated that there was no significant difference in the efficiency of neural communication under the three conditions. These results demonstrated that MVF combined with vibrotactile stimulation could strengthen the embodiment perception with increases in motor cortical activation, which indicated an evidence-based protocol of MVF to facilitate the recovery of patients with stroke.

## Introduction

Mirror visual feedback (MVF) is widely used in the field of upper limb and hand rehabilitation as a low labor-intensive, affordable, and convenient method (Wu et al., 2013; Samuelkamaleshkumar et al., 2014; Hebert et al., 2016). Recent studies reported that MVF was an evidence-based effective treatment to promote the recovery of motor functions, especially for upper limbs, and enhance the abilities of daily life in stroke patients (Pollock et al., 2014; Ding et al., 2018, 2019b; Thieme et al., 2018) from the reflection of the unaffected hand movements. MVF could prompt the multisensory integration of patients and contribute to balance the conflict between motor output and visual/proprioceptive feedback, whereby it makes patients embody the reflection, especially through experiencing the kinesthesia illusion (Ramachandran and Rodgers-Ramachandran, 1996; Altschuler et al., 1999). As a visual input dependent treatment, the sense of embodiment arising from MVF is recognized as one of the determinants for the efficacy of this treatment (Longo et al., 2008; Brunetti et al., 2015; Chancel et al., 2017). However, recent studies have taken little consideration of the influence of the subject's variability in embodiment and effective strategies to enhance the experience of embodiment, which might result in various findings and hinder the development of MVF.

Embodiment, which is also called bodily self-consciousness, is a type of experience, comprising a perception of body ownership, location, agency, and deafference (Longo et al., 2008; Blanke et al., 2015). It plays a critical role in mental life, closely relating to the sense of self (Longo et al., 2008). Studies suggested that embodiment has the potential to alter patients' sensorimotor activity and multisensory integration (Michielsen et al., 2011; Saleh et al., 2014; Medina et al., 2015; Ding et al., 2019a). Wainer et al. reported that embodiment could affect patients' engagement of robot-supported training and suggested a positive correlation between the experience of embodiment and effectiveness of treatment (Wainer et al., 2007). The perception of embodiment relies upon multisensory feedbacks (Medina et al., 2015; Azanõn et al., 2016). Visual input is recognized as the origin of embodiment (Pavani and Zampini, 2007; Ramachandran and Altschuler, 2009; Deconinck et al., 2015). In MVF, visual feedback could generate illusions, such as kinesthesia illusion and referred sensation, and induce the perception of the embodiment. Proprioceptive information plays a critical role in motor execution and control. Studies demonstrated that bimanual movements in MVF, where visual and proprioceptive feedbacks were involved, could enhance the perception of embodiment (Medina et al., 2015; Wittkopf et al., 2017). Furthermore, our previous study found that the combination of auditory and visual-proprioceptive feedback could facilitate facial embodiment in patients with Bell's palsy, which was parallel to Radziun's finding while using auditory cues in rubber hand illusion (Radziun and Ehrsson, 2018; Ding et al., 2020). These above studies indicated a positive correlation between sensory inputs and perception of embodiment, which suggested potential strategies enhancing embodiment.

In our previous study, a vibrotactile stimulation was employed to induce kinesthesia illusion for better motor imagery BCI control (Yao et al., 2015). Kinesthesia illusion is a kind of illusory proprioceptive experience without actual joint movement. Mechanical vibration and tactile stimulation of muscle tendon could evoke kinesthesia illusion, which could strengthen proprioceptive feedback in MVF. Therefore, we speculated that the combination of motor task with vibrotactile stimulation in MVF would strengthen proprioceptive inputs, promote kinesthesia illusion of the static hand, and enhance the perception of embodiment. Furthermore, study demonstrated that combining MVF and tactile sensory inputs could strengthen amputees' awareness of phantom limb (Hunter et al., 2003; Wittkopf et al., 2017). It reported that dual percepts evoked when a light touch was applied to one limb in MVF and referred to the missing limb. According to graded motor imagery, MVF is recognized as a visual induced motor imagery, which may contain components of motor and sensory experiences (Moseley, 2006; Voisin et al., 2011). As a type of visual induced imagery, MVF could generate referred sensations, where sensory stimulus evoked from one hand is referred to as the contralateral one behind the mirror (Ramachandran et al., 1995; Takasugi et al., 2011; Katsuyama et al., 2018). Therefore, we speculated that the vibrotactile sensory stimulus itself and the strengthened proprioceptive feedback could be referred to the dominant side via MVF and contribute to enhance embodiment. However, there are few studies investigating the influence of vibrotactile stimulation on embodiment in MVF, and to the best of our knowledge, no studies have explored its effect on the activation of the cortical area.

In this study, the tendon vibrotactile stimulation was combined with MVF to investigate its effects on the perception of embodiment in healthy subjects and to explore the alterations of cortical activities from the perspective of embodiment, which would provide scientific evidence for therapeutic protocol developments in the future, especially for sensorimotor rehabilitation.

## Methods

### Study Design and Participants

Twelve healthy subjects participated in the experiment (20–33 years with an average age of 25 years; 4 females and 8 males; 10 right-handed and 2 left-handed). None of the subjects had previously participated in studies or experiments on MVF. All subjects signed informed consent forms prior to the experiment. This study was in accordance with the Declaration of Helsinki and approved by the Research Ethics Committee of the University of Waterloo (ORE# 22900).

### MVF and Vibrotactile Stimulation

**Figure 1:**
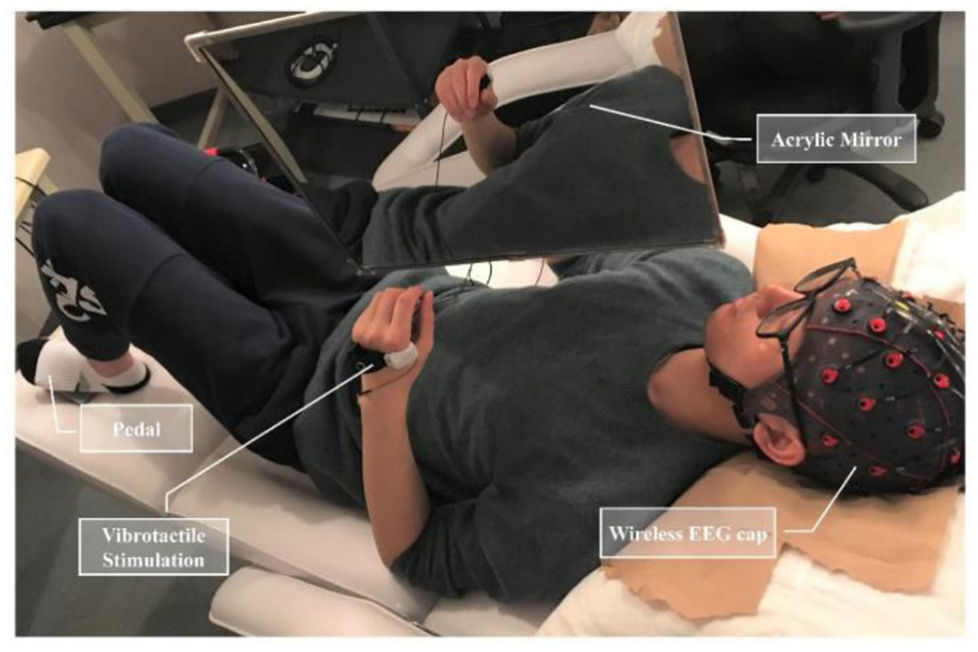
Experimental apparatus included a hanging acrylic mirror to provide mirror visual feedback, a wireless EEG cap, an actuator to present vibrotactile stimulation, and a foot pedal placed under the non-dominant foot.

In this experiment, a customized mirror holder was employed to mount a 40 cm by 50 cm acrylic mirror. The mirror was positioned over the chest area of a supine subject and could be adjusted at various angles (−90 to +90 degrees) in the sagittal plane (Figure 1). This device enabled an appropriate positioning of the mirror over a subject, who was required to place both upper limbs same position with the dominant arm behind the mirror and the other one on the reflecting side.

Vibrotactile stimulation was applied around the first interosseous dorsal muscle tendon of the non-dominant side. A liner resonance actuator (type C10-100, Precision Microdrivers Ltd.) sewn inside an elastic band was employed to provide vibrotactile stimulation. The actuator produced a 27 Hz sine wave modulated with a 175 Hz sine carrier wave, which can stimulate Pacinian corpuscles and Meissner corpuscles for the rich tactile experience (Yao et al., 2014). The amplitude of vibration was individually adjusted within the range of 0.5 times to maximum amplitude (11.3 μm) at the resonant frequency. The optimal amplitude was adjusted based on the feedback of the subjects, such that they could feel the vibration clearly and concentrate on performing the experimental tasks. In this experiment, there were three conditions corresponding to three types of stimulation, including no vibration (NV) as the control condition, continuous vibration (CV), and intermittent vibration (IV, alternated between 1-s stimulation with and 1-s rest).

### Experiment Paradigm

Subjects were instructed to lie down on a bed, place their non-dominant hand on the reflecting side of the mirror, and concentrate on the reflected hand in the mirror. In the experiment, they were required to keep their dominant hand still, perform non-dominant hand closing and opening (four fingers touching thumb and opening) at an approximate pace of 1 Hz, and keep all movements and facial expressions to a minimum. The setting was for the scenario of the dominant hand rehabilitation for its dysfunction affecting the lives of the patients more severely. A foot pedal was placed under the non-dominant foot. Pedaling was required as soon as subjects successfully perceived the sense of embodiment. Auditory cues (lasting 0.5 s) were provided to guide subjects to complete the task.

**Figure 2:**
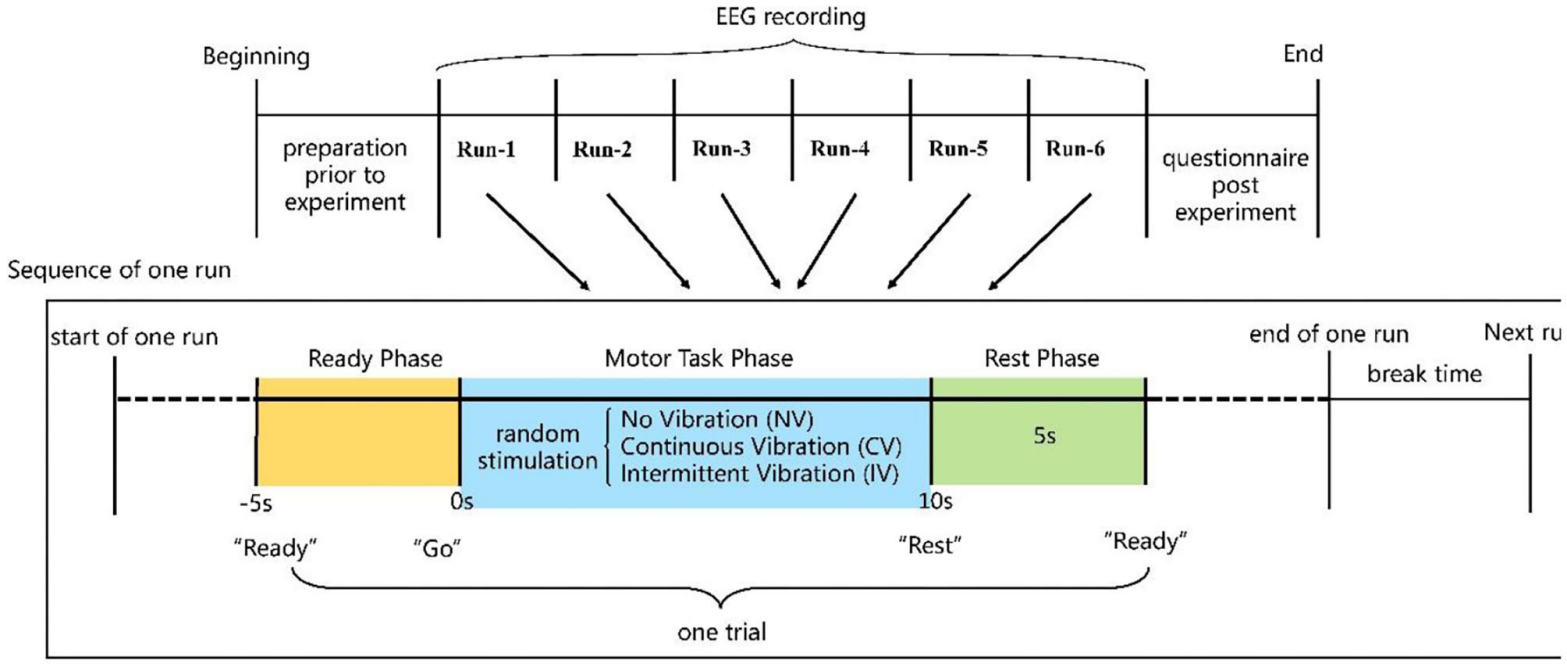
Experimental paradigm and sequence of events in each trial.

The experimental session comprised six runs of continuous EEG recording. In each run, subjects performed 30 trials for a total of 180 trials and rested between two runs. In each trial, subjects were prompted to perform non-dominant hand motor tasks following the cue while NV, CV, and IV stimulations were randomly applied. The sequence of events in each trial was illustrated in Figure 2. At −5 s (the start of each trial), auditory cue (“ready”) was provided to indicate the ready phase, during which subjects needed to concentrate and prepare to conduct the subsequent motor task. At 0 s, auditory cue (“go”) appeared, which indicated the beginning of the motor task. Subjects performed the motor task for 10 s and pedaled if the sense of embodiment was experienced. Each run contained 10 trials of NV, CV, and IV, respectively, and in random order. Each type of vibration stimulation lasted for 10 s. At 10 s, auditory cue (“rest”) appeared indicating the 5-s rest phase.

### Behavioral Measurements

Latency time (LT), which was defined as the period between the beginning of each trial of a motor task and when the pedal was tapped during that trial, was calculated to evaluate the ability of the investigated three experiment conditions to induce the embodiment perception. All the trials would be included in the computation of LT except for those where the subject did not pedal. For each subject, there were 24.2 out of 180 trials (13.4%) without pedaling, of which 10.1 trials were in NV, 7 trials were in CV and 7.1 trials were in IV.

Moreover, to assess the experience of mirror illusion and to evaluate the effects of three conditions on the perception of embodiment, the embodiment questionnaire (EQ) was completed by each participant after the experiment. The experience of embodying the mirror reflection, as one type of perception of altered ownership, was evaluated using a modified EQ based on previous studies (Botvinick et al., 1998; Longo et al., 2008; Wittkopf et al., 2017). EQ contained the location of a body part (L-1 “*It feels as if my hand is in the same location as the reflection of the hand*,” L-2 “*It seems like the reflection of the hand is in the location where my hand is*”), ownership of the reflection (O-1 “*It feels like I am looking directly at my hand rather than at a reflection of the hand*,” 0–2 “*It feels as if the reflection of the hand is part of my body*”), agency of the reflection (A-1 “*It feels as if I could move the reflection of the hand without having to move my dominant hand*,” A-2 “*It seems that if I move my dominant hand, the reflection of the hand will move too*”), and deafference (D-1 “*It feels like I cannot tell where my dominant hand is*,” D-2 “*My dominant hand feels unusual*”). Subjects were required to rate each statement for three different types of stimulation in random order using an 11-item Likert scale. “−5” represented “strongly disagree” with the statement, and “+5” indicated “strongly agree.”

### EEG Recording and Preprocessing

EEG signals were recorded using a 32-channel wireless EEG system (g.Nautilus, g.tec, Austria). Electrodes were placed according to the extended 10/20 system. The reference electrode was located on the right earlobe, and the ground electrode was located on the forehead. A hardware notch filter at 60 Hz was used, and signals were digitally sampled at 250 Hz. EEG signals of all subjects were fully checked to confirm the stability before and during the experiment.

EEG data were corrected before the analysis of event-related spectral perturbations (ERSP) or event-related desynchronization/synchronization (ERD/ERS). The signals were first inspected visually and the trials with the artifacts such as large drifts and electrode spikes were removed. After that, independent component analysis (ICA) was employed on the remaining trials to remove the artifacts from eye movements, blinks, muscle activities, etc. The average number of trials removed for artifacts was 20.1 out of 180 trials (11.1%), and the average independent components (ICs) removed per subject was 6.6.

The affected side of the patients in the clinic is possible for both the left and right sides. As such, both left-handed and right-handed subjects were recruited in this study for the potential clinical application in the future. For the consistency of the analysis, the EEG data from the subjects who were left-handed were flipped, such that channel C4 was defined as the non-dominant side (active), and channel C3 was defined as the dominant side (static). The first 4 s data of embodiment elicited was analyzed. The average number of trials where the embodiment perception was induced but <4 s after artifacts removal was 28.7 out of 180 trials (15.9%) per subject, of which NV, CV, and IV were 11.9, 8.4, and 8.3, respectively. The remaining number of trials was 110.9 per subject, of which NV, CV, and IV were 33.1, 39.2, and 38.6, respectively.

#### Event-Related Spectral Perturbations (ERSP)

ERSP visualizes the change of spectral power relative to the baseline. In this study, the resulting ERSP visualized the cortical responses of the left and right hemispheres to the embodiment of reflected hand in the mirror. The baseline interval was taken from −0.9 to −0.1 s, which was prior to the onset of the motor task lasting for 0.8 s. Before the spectral transformation, the small-Laplacian filter was applied to the preprocessed EEG data to accentuate the localized activities and increase the signal-noise ratio. A 0.8-s long sliding window was applied to segment the first 4 s data of embodiment during MVF. The step was 0.004 s. Fourier transform was conducted on each segment, and the spectra were normalized by dividing by their respective mean baseline spectra. The normalized spectral amplitude was log transformed (20log10) to represent power decreasing with negative values and increasing with positive values compared to the baseline. In this study, the ERSP at Channels of C3 and C4 were calculated, respectively.

#### Event-Related Desynchronization/Synchronization (ERD/ERS)

ERD/ERS displayed the cortical rhythm amplitude suppression or enhancement of brain regions with respect to a baseline reference. In this study, both the alpha (8–13 Hz) and beta (13–26 Hz) frequency bands were investigated. Same as the ERSP calculation, the small-Laplacian filter was applied to the preprocessed EEG data. The baseline reference interval was from −0.9 to −0.1 s. For the calculation of ERD/ERS, the data was first bandpass filtered, i.e., 8–13 and 13–26 Hz for the alpha and beta frequency band, respectively. A 0.8 s sliding window was used to segment the data. The step was 0.004 s. The amplitude of the samples within the window was squared and averaged. The ERD/ERS value was obtained by dividing by their baseline value after subtracting the baseline. In order to investigate the alterations of hand area activities, we calculated the ERD/ERS of Channel C3 and C4 over the embodiment period, respectively. Moreover, the ERD/ERS topography was also displayed to explore the underlying neural alterations among the three conditions.

#### Network Analysis

Network analysis reflected the efficiency of neural communication (Rubinov and Sporns, 2010). For the construction of an EEG network, in this study, the nodes of the network were the recording electrodes of EEG data and the weight of the connection between two nodes was the phase lag index (PLI) of the two EEG signals (Stam et al., 2007). The network sparsity was the ratio of the number of existing connections to all possible connections. The sparsity from 0.1 to 0.2 with an interval of 0.02 was studied to decrease the false positives from the uncertainty of the weak link. Two commonly used metrics, weighted clustering coefficient (wCC), and weighted shortest path length (wsPL), were employed to quantify the properties of an EEG network in this study. Same as ERD/ERS analysis, the alpha-beta frequency band (8–26 Hz) was studied. The wCC measured the local efficiency of network communication, and the wsPL measured the global efficiency of a network. The details of calculating the two metrics were described in Wang et al. (2010) and Holmes et al. (2004).

### Statistical Analysis

The underlying model assumptions were thoroughly checked by the Shapiro-Wilk's test for normality of distribution, and the Levene's test for the homogeneity of variances. Separate one-way analysis of variance (ANOVA) was conducted with LT, ERSP, and ERD/ERS value as response variables, respectively. The three conditions (NV, CV, and IV) were the three levels of the single factor (stimulation methods), and the subject was regarded as a random factor. The null hypothesis was these three conditions did not have a significant effect on the values of the LT, as well as the change of brain regions (ERSP and ERD/ERS). *Post-hoc* comparisons were performed with a Bonferroni correction. The Friedman test was used to test the effects of three different conditions on embodiment perception from EQ results. The data of EQ were then further analyzed *post-hoc* using the Wilcoxon signed-rank test with Bonferroni adjustment. The significant level was set at 0.05 with a two-sided test.

## Results

All the subjects experienced a moderate to a strong sense of embodiment and most of them preferred IV and demonstrated stronger embodiment within IV than the other two conditions.

### Latency Time

The LT under the three conditions was 4.4 ± 2.0, 3.7 ± 2.0, and 3.6 ± 2.1 s for NV, CV, and IV, respectively. Statistical analysis showed that the LT of NV was significantly longer than that of CV and IV (CV vs. NV, *p* < 0.01; IV vs. NV, *p* < 0.01). However, there was no significant difference in LT between CV and IV (*p* > 0.05).

### Event-Related Spectral Perturbation (ERSP)

**Figure 3:**
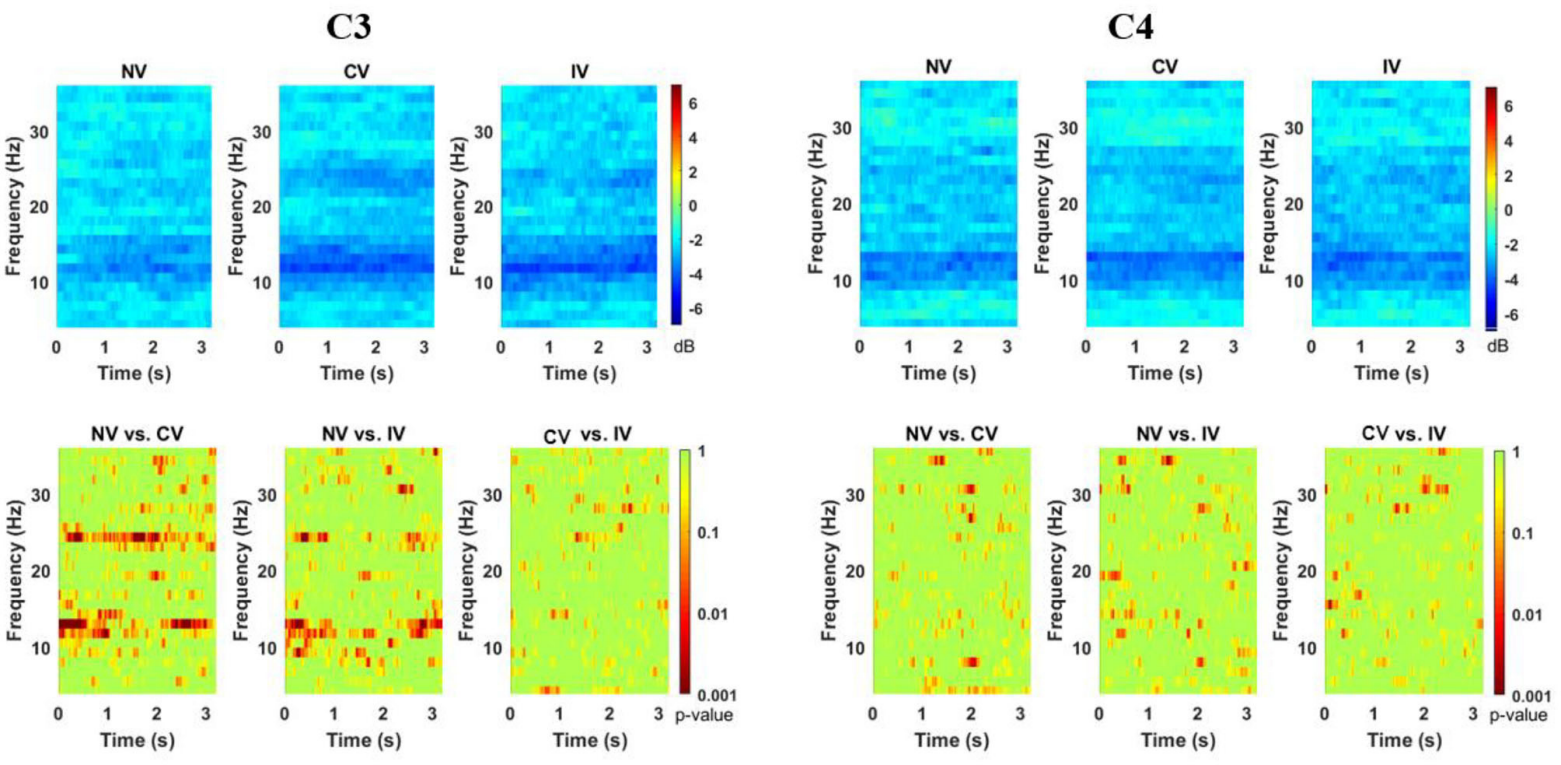
Changes of ERSP over embodiment experience for corrected channel C3 and C4 under the three conditions. The ERSP values were averaged across subjects. The bottom row showed the map for the statistical tests (*p*-value for the *post-hoc* comparisons). There were two discriminative power patterns at C3 in the range from around 10–15 Hz and 22–26 Hz in C3 under CV and IV conditions. C3 represented the dominant hand area and C4 represented the non-dominant hand area. NV, no vibration; CV, continuous vibration; IV, intermittent vibration.

The ERSP of the three conditions at C3 and C4 channels is shown in Figure 3. The desynchronization was observed in the alpha-beta frequency band (8–26 Hz) over the entire embodiment period. At channel C4, which corresponded to the non-dominant hand (active), the desynchronization was observed centering in the high-alpha (10–15 Hz) for all the three conditions, as expected. Interestingly, at channel C3, which corresponded to the dominant hand (static), the desynchronization was centered in two frequency bands, the high-alpha and high-beta (22–26 Hz) for the condition of CV and IV. Moreover, the desynchronization was more pronounced in high-alpha than in high-beta. For the condition of NV, the prominent desynchronization at C3 was only observed in the high-alpha frequency band. The statistical test showed that there were significant differences among the three conditions (*p* < 0.001) at C3. *Post-hoc* comparisons indicated that under the condition of CV and IV, the power of C3 in the high-alpha and high-beta frequency band was significantly lower than NV. Moreover, this tendency existed over the entire embodiment period. No consistent difference was found between CV and IV. For channel C4, there was no consistent significant difference among three conditions.

### Event-Related Desynchronization/Synchronization (ERD/ERS)

**Figure 4:**
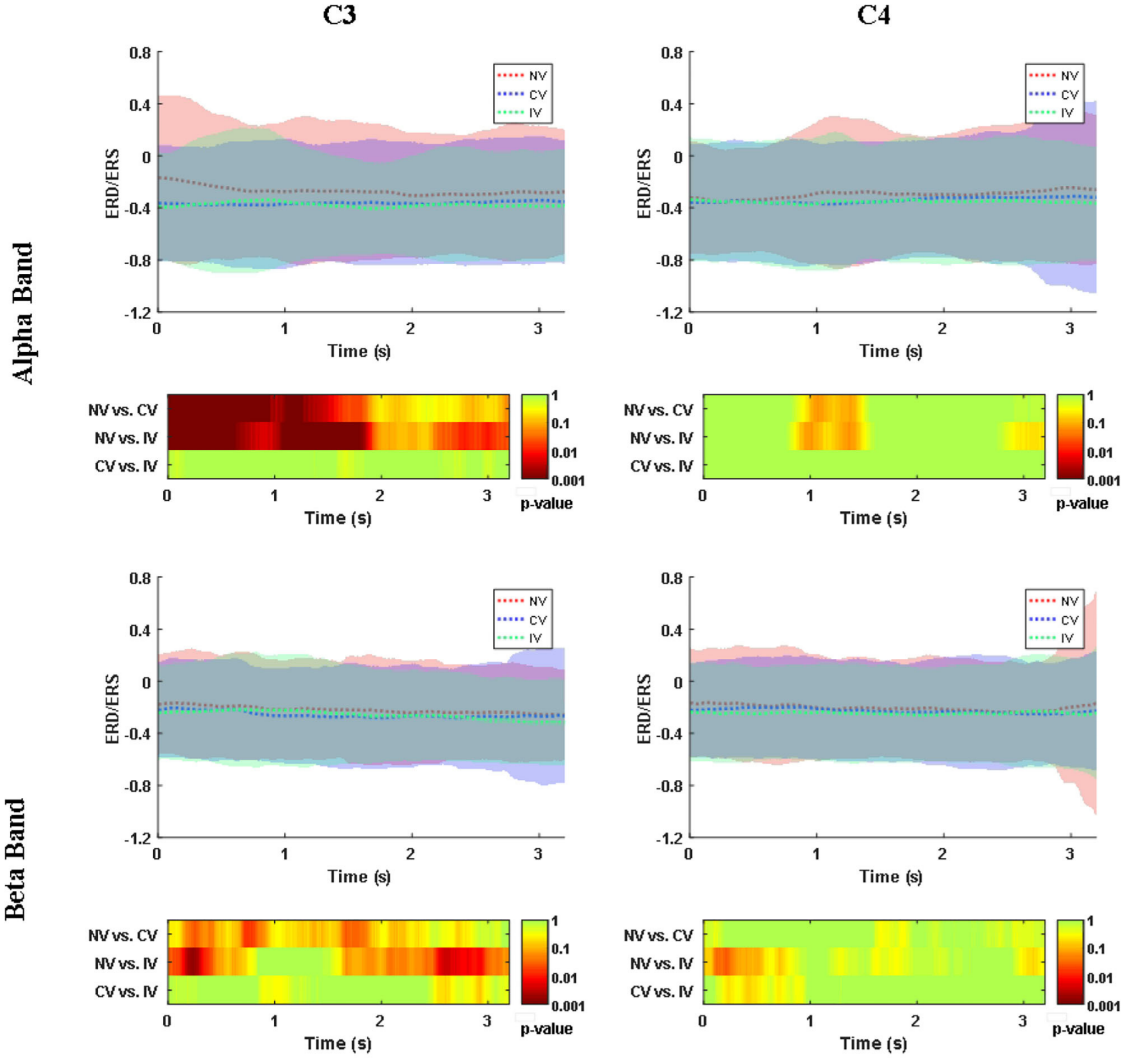
Changes of ERD/ERS in alpha (8–13 Hz) and beta (13–26 Hz) band over embodiment experience for corrected channel C3 and C4 under three conditions. The shading area around the dashed line indicated the averaged ERS/ERS ± SD. The values were averaged across subjects. The second and fourth row demonstrated the *post-hoc* comparison for the statistical tests (*p*-value). The value of NV in the alpha band was significantly higher than the values of CV and IV at C3. C3 represented the dominant hand area and C4 represented the non-dominant hand area. NV, no vibration; CV, continuous vibration; IV, intermittent vibration.

Figure 4 showed the ERD/ERS in alpha (8–13 Hz) and beta (13–26 Hz) frequency band over the embodiment period under the three conditions. ERD (power decrease with respect to baseline) was observed at channel C3 and C4 for both frequency bands of all the three conditions. For the alpha band, at C3, there was an ~30% power reduction compared to baseline under the NV condition, while the reduction was ~40% under the condition of CV and IV. At C4, the power reduction of all the three conditions was similar, around 40%. For the beta band, at C3, the power reduction of NV was ~22%, which was slightly higher than that of CV and IV. At C4, the power reduction of the three conditions was around 25%. Statistical analysis showed that the consistent significant difference between the experimental condition (CV and IV) and the control condition (NV) was only observed in the alpha band of C3, where the ERD of CV and IV was significantly stronger than that of NV over a large portion of the embodiment period (*p* < 0.05), approximately from the beginning to 2 s. In the beta band of C3, there were short periods lasting around 0.2 s when the ERD of CV and IV was significantly stronger than that of NV. For the comparison between CV and IV, no significant difference was observed in either frequency bands and channels.

**Figure 5:**
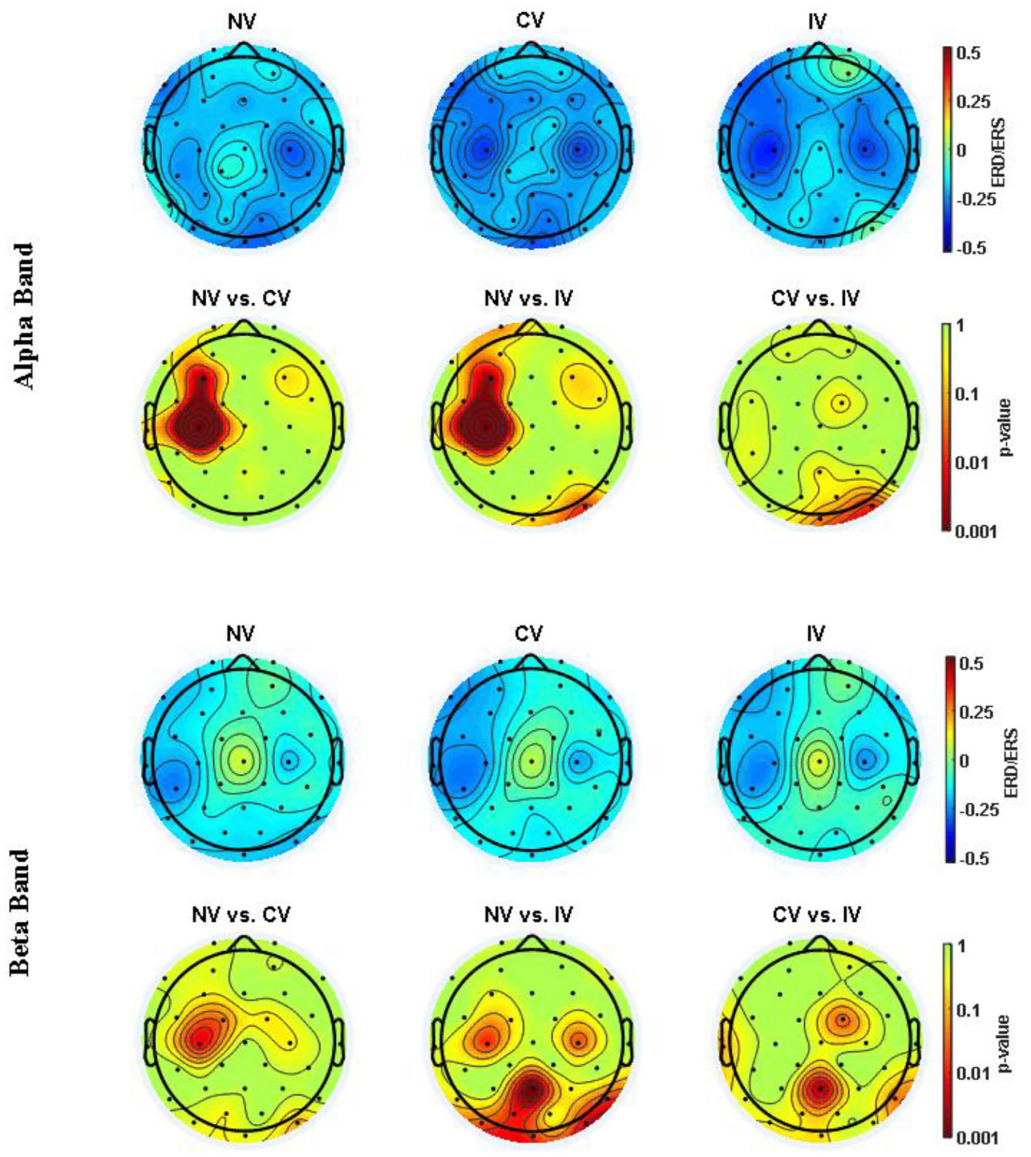
ERD/ERS topography in alpha (8–13 Hz) and beta (13–26 Hz) band averaged over embodiment experience for three conditions. The values were averaged across subjects. The second and fourth row showed the *post hoc* comparisons for the statistical test (*p*-value). NV, no vibration; CV, continuous vibration; IV, intermittent vibration.

In order to investigate the space-varying power alterations in different brain areas, the distribution of ERD/ERS value among the 32 electrodes were displayed in Figure 5 for both alpha and beta frequency bands. In the alpha band, ERD centering around the region at C3 and C4 was observed for all conditions. The desynchronization in the central-frontal region (C3 and F3) of the left hemisphere under the condition of CV and IV was more prominent than NV, where the difference was significant (*p* < 0.05). ERD around the parietal-occipital region (PO4, OZ) was also observed under the condition of NV and CV, while the desynchronization of IV in this area was slightly weak. In the beta band, the ERD was observed in similar regions, but weak compared to the alpha band. The desynchronization of CV and IV around the central region (C3) of the left hemisphere was significantly pronounced compared to that of NV (*p*<*0.05*). In addition, the desynchronization of IV was significantly pronounced around the right central (C4) and frontal-central region (FC2) compared to NV and CV, respectively (*p* < 0.05). In the parietal-occipital region, the ERD was not strong compared to that of the alpha band. The desynchronization was relatively strong under the condition of NV and CV compared to IV, especially at Pz.

### Network Analysis

**Figure 6:**
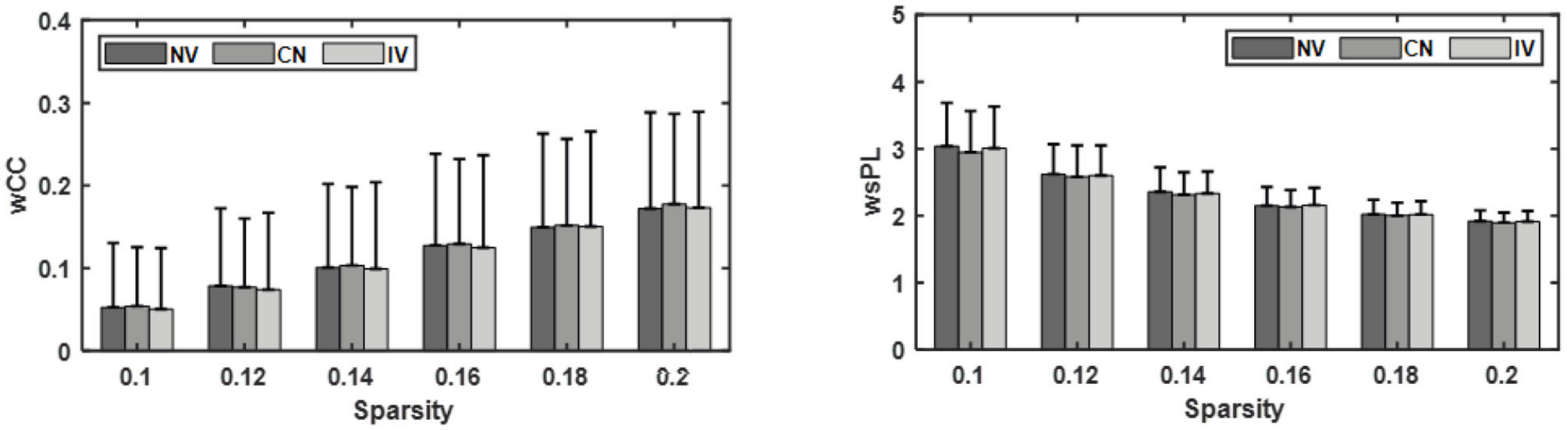
Comparison of the weighted clustering coefficient (wCC) and weighted shortest path length (wsPL) under three conditions: no vibration (NV), continuous vibration (CV), intermittent vibration (IV).

The wCC and wsPL with different sparsities were displayed in Figure 6. The difference among the three conditions was small. The wCC and wsPL values of CV were slightly higher and lower than that of the other two at the same sparsity, respectively. However, they were not significant in statistics. The instant effect of MVF and the transient embodiment perception alteration might be one possible reason.

### Embodiment Questionnaire

**Figure 7:**
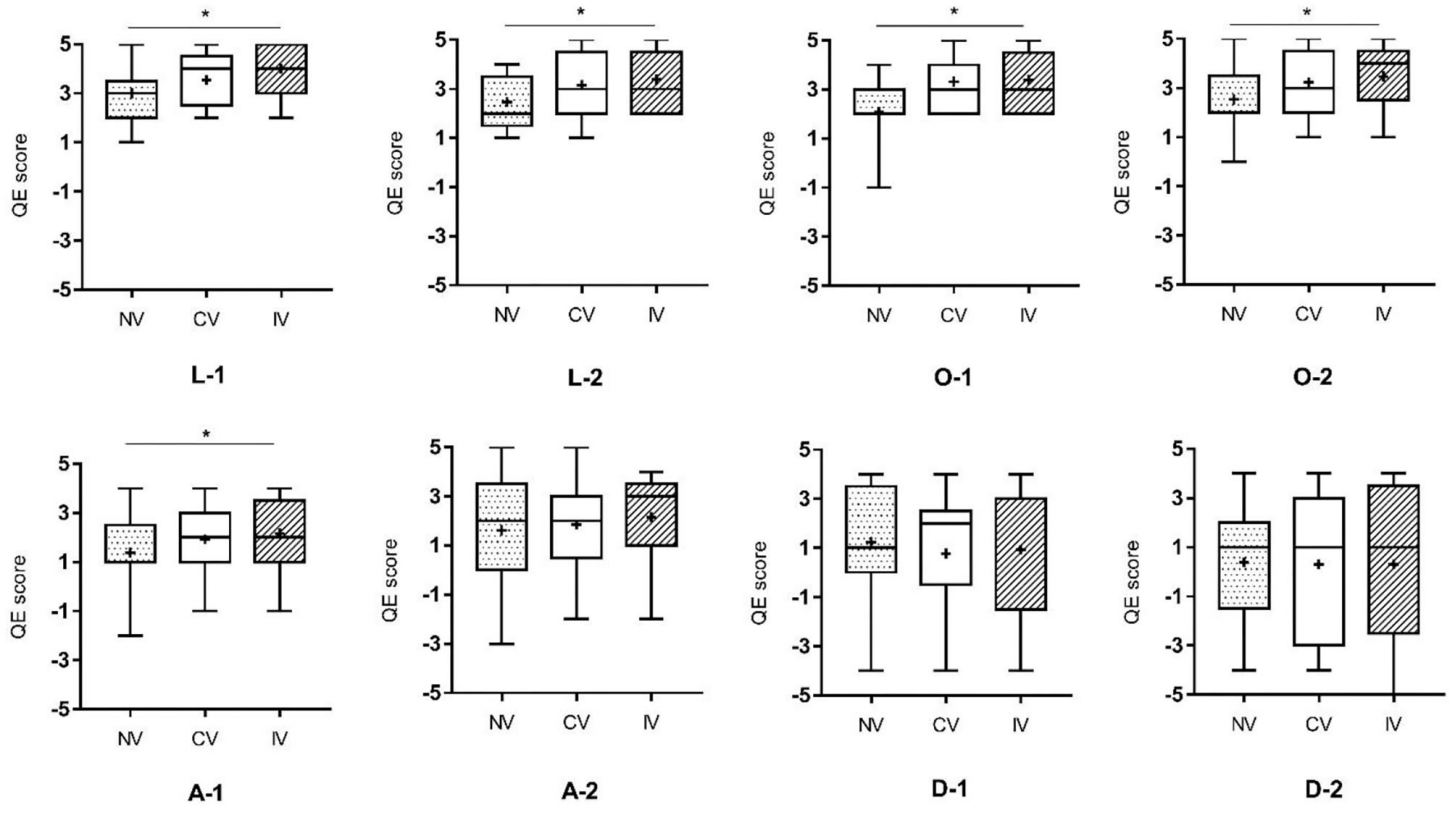
Results of Friedman test on the experience of embodiment among the three conditions. L-1, L-2: the two statements related to the location of a body part; O-1, O-2: the two statements related to ownership; A-1, A-2: the two statements related to agency; D-1, D-2: the two statements related to difference. NV, no vibration; CV, continuous vibration; IV, intermittent vibration. *p < 0.05.

The results of EQ were displayed in Figure 7. Friedman test among the three conditions showed that there were significant differences in statements on location, ownership, and the first statement of agency (*p* < 0.05: L-1, L-2, O-1, O-2, and A-1; *p* < 0.001: O-1). *Post-hoc* analyses suggested that significantly higher scores of statements for IV compared to NV were found on L-1, L-2, O-1, O-2, and A-1. Higher scores of statements for CV compared to NV were found on L-2, O-1, and O-2. However, no significant difference in the scores of EQ was obtained between CV and IV.

## Discussion

This study provides tentative evidence that MVF, when combined with vibrotactile stimulation (both continuous and intermittent stimulation), could enhance the perception of embodiment in healthy subjects. Moreover, according to the best knowledge of the authors, this study is the first to investigate the relating alterations of cortical activities. The results indicated that the integration of these two sensory inputs could strengthen embodiment experience with motor cortical activation increasing.

### Enhancement of Embodiment

Mirror visual feedback could make subjects embody the reflected hand in the mirror via visual inputs only or combining with proprioceptive feedback to promote the sense of embodiment (Ramachandran et al., 1995; Altschuler et al., 1999; Holmes et al., 2004; Wittkopf et al., 2017). Tendon vibrotactile stimulation could evoke kinesthesia illusion and was employed in our previous study to strengthen proprioceptive feedback (Yao et al., 2015). In the present study, we found that combining MVF with vibrotactile stimulation could better reduce the induction time of embodiment and strengthen the degree of subjective experience, comparing with pure MVF. According to our previous study, vibrotactile stimulation could strengthen proprioceptive feedback and provide tactile input. Thus, one possible interpretation might be the combination of motor task and tendon vibrotactile stimulation induced kinesthesia illusion strengthens proprioceptive feedback and contributes to enhance the sense of embodiment, comparing to pure motor task in MVF.

Another potential interpretation might be the interactions of referred sensations evoked by MVF. Medina et al. suggested that multisensory integration using MVF could promote the subjective embodiment experience (Medina et al., 2015). Our previous study also showed facial MVF with enunciation task, where three sensory modalities interacted, could facilitate facial embodiment (Ding et al., 2020). Besides, EEG and fMRI studies reported that referred sensations could activate the somatosensory cortex (Taylor-Clarke et al., 2002; Schaefer et al., 2009). Thus, the interaction of referred vibrotactile and proprioceptive stimuluses, and visual feedback might be another potential interpretation for the enhancement of embodiment with the combination of MVF and vibration. Embodiment is recognized as one determinant of the efficacy of mirror therapy, which might influence the treatment outcomes [33]. Our findings, which showed the enhancement of embodiment by combining MVF and vibrotactile stimulation, might contribute to developing a more effective MVF training protocol in the future. This inference is supported by the results of Lin et al., a similar study (Lin et al., 2014). They reported that the combination of afferent stimulation of hand and MVF could reduce motor impairment of the upper limb and improve daily function, especially for manual dexterity in patients with stroke. Moreover, referred sensations induced by MVF and tactile stimulation involve the activities of the somatosensory cortex, which might also facilitate rehabilitation (Schaefer et al., 2006).

Our study demonstrated that both continuous and intermittent vibration could enhance embodiment experience and there was no significant difference in the effect between these two stimulations. However, higher scores of EQ were obtained when subjects received IV rather than NV, which suggested a trend of enhancement in the embodiment under the condition of IV. According to the feedbacks from subjects, IV provided tactile stimulation and acted as a metronome in the experiment providing vibratory cue, which might prolong the sensory perception and strengthen subjective embodiment experience.

### Alterations of Brain Activities

The alpha band rhythms demonstrated ERD in memory and movement tasks (Pfurtscheller and Lopes Da Silva, 1999). High alpha desynchronization, also named mu-rhythm suppression, occurred in the sensorimotor related regions when performing goal-oriented exercise or observation (Bae et al., 2012). In our present study, ERD, as well as ERSP, revealed the desynchronization of the high alpha band in the dominant hand area under MVF. The desynchronization was significantly strengthened when combined with vibration. These findings provided electrophysiological evidence for the capability of vibrotactile stimulation in facilitating motor cortical activity, which might contribute to motor recovery in patients with stroke. In the beta band, the ERD of the two vibration conditions was stronger than the pure MVF in some short periods. It is reported that a beta ERD localized close to hand areas occurred when there was motor imagery of hand movement (Pfurtscheller et al., 1997). Moreover, Dockstader et al. demonstrated that selective attention to somatosensory stimulation could strengthen beta ERD in the primary somatosensory cortex (Dockstader et al., 2010). This might be one possible interpretation of our findings in the study, where subjects received vibration stimulation and were required to imagine their dominant hand moving while watching the reflected non-dominant hand.

Comparing to pure MVF, a stronger sense of embodiment was obtained when the subjects received vibrotactile stimulation in our study, and meanwhile more prominent motor cortex activation was observed. As a visual input based priming technique, MVF presented the ability to upregulate the activity of the motor system, visual cortex, and intercortical circuitries, which revealed the benefits for motor recovery (Wasaka and Kakigi, 2012; Mehnert et al., 2013; Franz et al., 2016; Inagaki et al., 2019). Moreover, some studies on MVF demonstrate increased activity in the posterior parietal cortex (especially for precuneus), dorsolateral prefrontal cortex, and insula (Fink et al., 1999; Dohle et al., 2011; Wasaka and Kakigi, 2012). Those brain regions play a prominent role in the sense of body ownership and bodily self-awareness (Tsakiris et al., 2007; Farrer et al., 2008; Karnath and Baier, 2010). Besides, the activities of the posterior insula and frontal operculum were thought to be related to body ownership in RHI (Tsakiris et al., 2007). In the present study, the ERD/ERS topography showed significantly pronounced desynchronization around the central-frontal region in the alpha band under the condition of IV and CV. We inferred that the enhancement of embodiment with increases in the activities of the motor region might be the result of the mediation of ownership related brain regions. However, as a limitation of the present channel-based EEG study, each node was assumed to represent the underlying brain region activation (Rubinov and Sporns, 2010). Thus, in the future study, other methods, such as fMRI, should be adopted to justify the relationship between cortical activation and embodiment experience and to show the potential mechanism of neural modulation.

The ERD/ERS topography also indicated an ERD localized around the parietal-occipital region when the subjects received NV and CV, especially in the alpha band. According to an fMRI study, supplementary activation was found in visual areas during MVF (Matthys et al., 2009). Our previous study also showed that after the intervention of MVF, an increase in communication efficiency was found in the visual area in stroke patients (Ding et al., 2019a). These might suggest visual inputs as a crucial basis for this approach. However, under the condition of IV, the ERD around parietal-occipital areas was not comparable with NV and CV, which was similar to the investigation of Yao et al. (2018). As suggested by Yao's finding, we inferred that the IV played a role as a vibratory cue in our study, which induced the somatosensory attentional orientation and influenced the desynchronization over the parietal-occipital region (Yao et al., 2018). Thus, we speculated there was another neural modulation pattern during IV which cognition accounted for.

### Limitation

As a pilot study, we focused on the period after inducing the embodiment, proving the effectiveness of the vibrotactile stimulation on enhancing the embodiment perception. The cortical activities from the beginning of the motor task to the acquirement of embodiment was not analyzed here due to a small number of subjects and the difference of latency time in subjects. A long-term follow-up study with a large sample size might contribute to explore the cortical alterations from the perspective of network connectivity. Moreover, the transition period will be investigated in future studies through subgrouping subjects with latency time.

## Conclusion

This study investigated the effect of combining MVF and vibrotactile stimulation on the perception of embodiment in healthy subjects. Moreover, we firstly used EEG to explore the related alterations of cortical activities. Our results revealed that MVF combined with vibrotactile stimulation had the ability to strengthen the perception of embodiment and promote motor cortical activities. Besides, this study provided an evidence-based protocol of MVF training, which might be applied to facilitate the recovery of patients with stroke.

## Data Availability Statement

The raw data supporting the conclusions of this article will be made available by the authors, without undue reservation.

## Ethics Statement

The studies involving human participants were reviewed and approved by the Research Ethics Committee of the University of Waterloo (ORE# 22900). The patients/participants provided their written informed consent to participate in this study and for the publication of any identifiable images or data.

## Author Contributions

JJ and NJ conceived the study. LD performed the experiment. JH and LD analyzed the data and wrote the manuscript. LY, SC, and HW revised the manuscript. All authors read and approved the final manuscript.

## Conflict of Interest

The authors declare that the research was conducted in the absence of any commercial or financial relationships that could be construed as a potential conflict of interest.
